# Severe, Non‐apneic Respiratory Dysfunction and Hypoxia following Generalized Convulsive Seizures

**DOI:** 10.1002/ana.78164

**Published:** 2026-01-27

**Authors:** Haley E. Pysick, Rup K. Sainju, Roshni Nair, Deidre N. Dragon, Eduardo Bravo, Laura Vilella, Xiaojin Li, Samden D. Lhatoo, George B. Richerson, Brian K. Gehlbach

**Affiliations:** ^1^ Department of Internal Medicine University of Iowa Iowa City IA; ^2^ Department of Neurology University of Iowa Iowa City IA; ^3^ Department of Neurology McGovern Medical School, University of Texas Health Science Center at Houston Houston TX; ^4^ Department of Neurology Hospital del Mar Barcelona Spain; ^5^ Iowa City VA Health Care System Iowa IA; ^6^ Iowa Neuroscience Institute, University of Iowa Iowa IA; ^7^ Department of Molecular Physiology and Biophysics University of Iowa Iowa City IA

## Abstract

**Objective:**

Sudden unexpected death in epilepsy (SUDEP) is a devastating consequence of some generalized convulsive seizures (GCS). Recent work has focused on seizure related apnea as a biomarker of SUDEP risk, frequently without characterizing the adequacy of non‐apneic ventilation or identifying other dysfunctional breathing patterns. We hypothesized that GCS frequently induce immediate, severe, non‐apneic respiratory dysfunction that can induce critical hypoxia and bradycardia and sought to characterize breathing patterns after GCS.

**Methods:**

Adult patients admitted to an epilepsy monitoring unit were studied. The effects of GCS on breathing and heart rate were analyzed using nasal pressure transducers, chest and abdominal respiratory inductance plethysmography, capillary oxygen saturation, transcutaneous CO_2_, electrocardiogram, electroencephalogram, and expert audiovisual analysis. Correlation analyses, the Mann–Whitney test, and an unpaired *t* test were used to analyze relationships between dysfunctional breathing patterns and both the severity of postictal hypoxemia and the heart rate.

**Results:**

Thirty‐two GCS from 22 patients were analyzed and 31 exhibited 1 or more of the following breathing patterns: disordered rhythmicity (n = 28/32, 87.5%), shallow breathing (n = 12/32, 37.5%), thoracoabdominal asynchrony (n = 24/30, 80.0%), and upper airway obstruction (n = 30/32, 93.8%). Oxygen desaturation was more severe when postictal breathing was shallow or irregular in amplitude. The latter was associated with absolute or relative bradycardia.

**Interpretation:**

Nonfatal GCS frequently induce immediate, severe, non‐apneic respiratory dysfunction temporally associated with severe hypoxia and bradycardia. Our study suggests that postictal respiratory and cardiac function are tightly coupled and highlights the importance of including all the relevant pathologic variables in studies of SUDEP pathogenesis. ANN NEUROL 2026;99:1263–1276

Sudden unexpected death in epilepsy (SUDEP) is the leading category of premature death in people with epilepsy (PWE).^1^ The MORTEMUS study documented changes in electrocardiograms (ECG) and respiratory rates in patients who died of SUDEP in epilepsy monitoring units (EMUs).^2^ In 9 of 11 SUDEP patients in whom both heart rate and respiratory rate could be measured, terminal apnea preceded terminal asystole, and all deaths occurred shortly after a generalized convulsive seizure (GCS). This sequence has since been replicated in multiple animal models of seizure‐induced death.^3^, ^4^, ^5^

MORTEMUS was important in identifying respiratory dysfunction as the primary cause of death in some cases of SUDEP. Still, controversy remains regarding some of its findings. For example, it is sometimes noted^6^, ^7^ that cardiac changes, principally bradycardia and transient asystole, occurred before apnea in a subset of individuals, suggesting that cardiovascular dysfunction may have been the initiating event in those cases. This interpretation does not account for certain limitations in MORTEMUS, in which breathing was not directly measured, but was retrospectively assessed through the analysis of video recordings and breathing artifacts on electroencephalography (EEG). Although the authors suggested that hypoventilation may have also occurred in the absence of apnea, and in some cases described findings consistent with dysfunctional breathing, the design of the study precluded detailed characterization of non‐apneic breathing patterns. Consequently, formal analyses of respiration focused on changes in the respiratory rate and the identification of 10‐second central apneas. Because measurements of CO_2_ or O_2_ were not made, and non‐apneic breathing patterns were not quantified, the possibility remains that some patients developed severe respiratory dysfunction and hypoxia that may have secondarily caused the observed cardiac changes.^4^

Investigation of this possibility is critical, because although the ability of seizures to induce severe hypoxia is well established,^8^, ^9^, ^10^ the optimal treatment of patients with bradycardia and/or hypoxia would depend on the cause of both the bradycardia (primary cardiac versus respiratory) and the hypoxemia. For instance, a patient with hypoxemia from upper airway obstruction would be expected in most cases to improve with repositioning, whereas a patient with central apnea would not. Similarly, although electrically pacing the heart would ameliorate postictal bradycardia, it would not improve ventilation or oxygenation.

We hypothesized that some seizures induce immediate, severe respiratory dysfunction and hypoxia and sought to characterize breathing patterns after GCS. We further hypothesized that respiratory dysfunction would modulate the heart rate and predispose to bradycardia. To test these hypotheses, we retrospectively analyzed all available respiratory data from a cohort of patients who underwent video‐EEG and comprehensive cardiorespiratory monitoring in our EMU. Here, we describe a variety of severe respiratory abnormalities in the immediate postictal period following nonfatal GCS that could have been missed in MORTEMUS by focusing on central apneas and would worsen the accrual of an oxygen debt that likely developed during the seizures.

## Patients and Methods

### Study Design and Participants

This study used data from prospectively enrolled patients admitted to the University of Iowa EMU between June 2015 and July 2023. Data from some participants have been reported previously as part of the multicenter Center for SUDEP Research or related studies.^11^, ^12^, ^13^, ^14^, ^15^, ^16^, ^17^, ^18^, ^19^, ^20^, ^21^, ^22^ Participants were 18 years or older and had 1 or more GCS recorded during their inpatient EMU stay. Exclusion criteria included a history of stroke, active cardiac or pulmonary disease, space‐occupying brain lesions, and known or potential pregnancy. Approval was obtained through the University of Iowa institutional review board. All participants provided written consent.

Demographic and clinical variables were obtained via written questionnaire and query of the electronic medical record (see Data S1). This report adheres to all elements of the STROBE checklist for observational studies (www.strobe-statement.org/checklists/).

### Video‐EEG and Cardiorespiratory Monitoring

All patients underwent continuous video‐EEG monitoring and peri‐ictal care as directed by the clinical service. Scalp EEG electrodes were placed according to the international 10 to 20 system with the addition of T1 and T2 electrodes. Multimodal polygraphy included 3‐lead ECG, peripheral capillary oxygen saturation (SpO_2_), and transcutaneous CO_2_ (tcCO_2_) measurement. Thoracic and abdominal excursions were recorded using respiratory inductance plethysmography (RIP) and airflow was assessed using a nasal pressure transducer (NPT) and oronasal thermistor (see Data S1).

All seizures were reviewed by 3 investigators (H.E.P., R.K.S., and B.K.G.) including a board‐certified epileptologist (R.K.S.) and board‐certified pulmonary and critical care physician (B.K.G.). Only seizures with evaluable O_2_/CO_2_ data from oximetry and/or tcCO_2_ data plus respiratory pattern data from appropriately positioned RIP belts and/or NPT were included. CO_2_/O_2_ data were analyzed as previously described (see Data S1).^19^ Duration of postictal generalized EEG suppression (PGES) was determined using an automated and validated tool^21^, ^23^ and supplemented by visual analysis when necessary. GCS ictal semiology was characterized as per Alexandre et al.^24^ Audio and video analysis was used to assess body position, upper airway sounds, accessory muscle use, and movement of the chest and abdomen; to confirm maintenance of stable positioning of the monitoring devices; and to identify artifact from muscle activity and nursing interventions.

### Cardiorespiratory Analyses

Postconvulsive breathing was analyzed in 2 stages. We first identified 4 breathing patterns through a comprehensive and iterative review of all audiovisual and signal data. Visual analysis of RIP belt waveforms was used to assess rate and regularity of breaths and to identify instances of thoracoabdominal asynchrony, defined as nonparallel movement of the thoracic and abdominal compartments. Konno‐Mead plots were constructed to better visualize synchrony of thoracoabdominal wall motion.^25^ Instances of postconvulsive central apnea (PCCA) were defined as 1 or more missed breaths without any other explanation (ie, movement or intervention) and of at least 5 seconds duration if occurring immediately after the end of the convulsive period.^16^ RIP belts were also used to qualitatively assess depth of respiration, which included the identification of grossly ineffective respiratory efforts or “shallow breaths.” The NPT waveform was used to detect airflow limitation in the inspiratory and expiratory phases^26^ and to help identify instances of shallow breathing and/or irregularity.

Next, we quantified disordered rhythmicity and shallow breathing during the first 60 seconds of the postconvulsive period (see Data S1). For disordered rhythmicity, we used a previously published method^11^ to analyze respiratory variability, which was expressed as the coefficient of variation of the inter‐breath interval (CoV‐IBI). To quantify the occurrence of shallow breathing, we determined the amplitude of each breath, expressed as a percentage of baseline amplitude. Seizures associated with 2 or more breaths smaller than baseline were considered to exhibit shallow breathing. Changes in respiratory amplitude over time were modeled using linear regression.

The association between postconvulsive respiratory dysfunction and heart rate was analyzed after calculating R–R intervals (RRI) from the same period (see Data S1). Instantaneous heart rates were calculated as 1/RRI × 60 beats per minute (bpm). To identify potentially clinically relevant instances of bradycardia we determined the minimum heart rate for each postconvulsive period using a 4‐beat moving average, with bradycardia defined as a heart rate <60bpm. Transient relative bradycardia was defined as an acute fall in heart rate by at least 40bpm to a value below 100bpm with rapid recovery to over 100bpm.

### Statistical Analysis

Our statistical analysis plan had 2 major components. The first was to characterize the observed respiratory patterns and relate them to the development of critical hypoxia. For this reason, we focused on SpO_2_ nadir as our primary outcome of interest. Secondary outcomes included the associations between breathing patterns and duration of hypoxemia and the development of postictal hypercapnia. The second major component of our analysis was to analyze the association between the observed postconvulsive breathing patterns and heart rate.

Some patients had more than 1 evaluable GCS. All seizures were pooled for descriptions of seizure characteristics. For inferential statistical analyses, however, only the first evaluable GCS for each patient was used. Data were summarized as mean (standard deviation [SD]) or median (interquartile range [IQR]) depending on their distribution. Spearman's ρ or Pearson's *r* was used to examine the relationships between tcCO_2_ and SpO_2_ and selected clinical variables. An unpaired *t* test or Mann–Whitney test was used to analyze differences between independent groups. The Wilcoxon matched‐pairs signed rank test was used to analyze differences between baseline and postconvulsive CoV‐IBI and heart rate. All tests were 2‐sided and *p* ≤ 0.05 was considered statistically significant. Correction for multiple comparisons was not performed because of the exploratory nature of the study.

## Results

### Patient and Seizure Characteristics

A total of 270 patients were enrolled, of whom 59 had 106 GCS. No seizures were fatal. Twenty‐two patients had 33 seizures with both (1) oximetry (n = 28) and/or tcCO_2_ data (n = 26) and (2) evaluable RIP belt (n = 31) and/or NPT (n = 15) data (see Data S1). One of these seizures (with SpO_2_, tcCO_2_, and RIP belt data) was subsequently excluded because the patient received extensive bag‐mask ventilation during the convulsive and postconvulsive periods.

Patient and seizure characteristics are shown in Table 1. All (32/32, 100%) seizures were associated with some form of nursing intervention including suctioning (27/32, 84.4%) and/or repositioning (21/32, 65.6%). In 9 seizures, oxygen was administered, and in the 6 seizures with an evaluable SpO_2_ this was performed 13.5 (−3.5 to 28.3) seconds after (and no sooner than 7 seconds before) the time of occurrence of the SpO_2_ nadir.

**TABLE 1:** Patient and Seizure Characteristics

Characteristics	N	Value (mean ± SD or median [IQR]%)
Age (yr)	22	35.1 ± 11.6
Sex (n, % female)	22	11 (50.0)
Duration of epilepsy (yr)	22	11.2 ± 9.7
Body mass index (kg/m^2^)	22	30.5 ± 8.2
Obstructive sleep apnea (%)	22	1 (4.5)
SSRI/SNRI (n, %)	22	6 (27.3)
GCS characteristics		
Total GCS (n, %)	32	32 (100)
Focal to BTC		29 (90.6)
Genetic GCS		2 (6.3)
Unclear		1 (3.1)
Seizure duration (s)	32	94.0 (76.0–143.0)
Convulsion duration (s)	32	59.0 (48.5–70.0)
GCS type^a^	31	
Type 1		4 (12.9%)
Type 2		3 (9.7%)
Type 3		24 (77.4%)
PGES duration (s)	32	31 (5.0, 41.0)
Postictal SpO_2_ nadir (%)	22	61.4 ± 16.5
Duration of O_2_ desaturation (s)	22	108 [54.8–172.0]
Peak postictal CO_2_ (mmHg)	21	57.2 ± 9.9
Postictal increase in tcCO_2_ (mmHg)	21	17.2 ± 5.6
Duration of increased postictal tcCO_2_ (s)	19	430.2 ± 233.7

^a^ GCS ictal semiology was characterized as per Alexandre et al.^21^ Unable to characterize 1 GCS because of incomplete video coverage.

BTC = bilateral tonic clonic seizure; GCS = generalized convulsive seizure; PGES = postictal generalized EEG suppression; SpO_2_ = capillary oxygen saturation; SSRI/SNRI = selective serotonin reuptake inhibitor/serotonin norepinephrine reuptake inhibitor; tcCO_2_ = transcutaneous carbon dioxide.

The respiratory rate in the first minute following GCS ranged from 5 to 40bpm with a median of 21.5 (19.3–24.8). All seizures (n = 32/32, 100%) demonstrated eventual tidal volume recruitment through a combination of increased inspiratory capacity (deeper inspiration) and active expiration, with video and RIP belt evidence of abdominal muscle activation below the interictal relaxation volume. Despite the modest increase in respiratory rate, hypercapnia was common after GCS, with a mean peak postictal tcCO_2_ of 57.2 ± 9.9mmHg in 21 GCS. Oxygen desaturation was also common and frequently severe, with SpO_2_ nadir <70% occurring in over half (13/22, 59.1%) of all evaluable seizures. The median time to SpO_2_ nadir (n = 22) and peak tcCO_2_ (n = 21) following the end of the convulsive period was 8.0 (0.8–19.5) and 143.5 (100.3–194.3) seconds, respectively.

In analyses restricted to the first evaluable GCS from each patient, respiratory rate was unrelated to tcCO_2_ peak (n = 17), magnitude of postictal increase in tcCO_2_ (n = 17), or duration of increased tcCO_2_ (n = 18; all *p* > 0.17). Severity of oxygen desaturation as expressed by SpO_2_ nadir was similarly not associated with respiratory rate (*r* = 0.035, *p* = 0.91, n = 13) or any of these tcCO_2_ variables (all *p* > 0.34). In contrast, SpO_2_ nadir was negatively associated with duration of oxygen desaturation (*r* = −0.78, *p* = 0.008, n = 10), while the latter was also positively associated with duration of tcCO_2_ elevation (*r* = 0.58, *p* = 0.039, n = 13). There was no relationship between SpO_2_ nadir and either vigilance state at seizure onset or duration of PGES (both *p* > 0.66) (see Data S1).

Of 32 seizures analyzed, 31 (96.9%) had evidence of 1 or more obviously or possibly dysfunctional breathing patterns. These patterns were classified into 4 categories: disordered rhythmicity, shallow breathing, thoracoabdominal asynchrony, and upper airway obstruction (UAO).

### Disordered Rhythmicity

**FIGURE 1:**
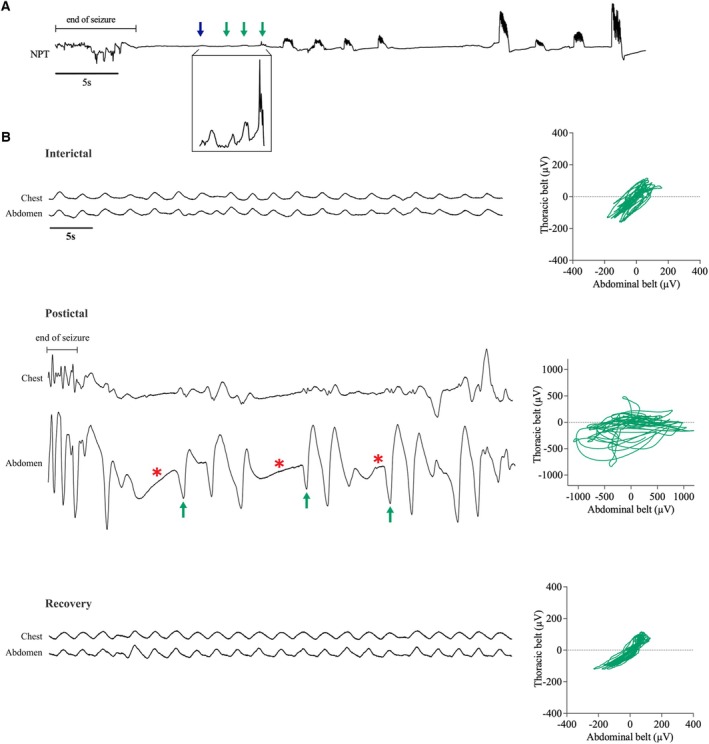
Examples of irregular breathing patterns in the immediate postictal period. (A) Overt PCCA lasting approximately 6 seconds with movement artifact (blue *arrow*), grossly ineffective efforts (green *arrows*), and additional shallow breaths before another 8‐second apnea occurs. Following this second apnea the tidal volume increased. This patient desaturated to 24% in the postictal period. This tracing also demonstrates high‐frequency inspiratory oscillations consistent with upper airway obstruction. (B) RIP belt tracings and Konno‐Mead plot in the interictal and postictal periods. The interictal breathing shows a regular rate and rhythm. In the postictal period, RIP belts demonstrate erratic and irregular patterning followed by shallow breathing that deepens with time. There are several instances of interrupted expiration in which passive expiration is followed by a pause (red *asterisks*), after which active expiration occurs (green arrows). PCCA = postconvulsive central apnea, RIP = respiratory inductance plethysmography. [Color figure can be viewed at www.annalsofneurology.org]

PCCA occurred in 3 of 32 seizures (9.4%) (Fig 1A). In addition, many patients exhibited respirations that did not meet criteria for PCCA, but which were irregular and/or chaotic (see Fig 1). Overall, respiratory variability (CoV‐IBI) increased from baseline during the first 60 seconds of the postconvulsive period, from a median of 10.8 (6.3–18.0)% to 26.5 (19.3–41.3)%, *p* < 0.0001, n = 22. In 2 of 32 seizures (6.3%), there was 1 or more higher amplitude breaths immediately following the convulsive period before breathing transitioned to a more rapid, irregular pattern. Two seizures (6.3%) not scored as PCCA exhibited a pattern of “interrupted” expiration, which consisted of an apnea interposed between passive and active expiration (see Fig 1B).

Postconvulsive CoV‐IBI was not significantly associated with SpO_2_ nadir (ρ = −0.52, *p* = 0.073, n = 13). There was also no relationship between postconvulsive CoV‐IBI and either peak postictal CO_2_ (ρ = −0.24, *p* = 0.35, n = 17) or duration of hypercapnia (ρ = −0.23, *p* = 0.37, n = 18).

### Shallow Breathing

**FIGURE 2:**
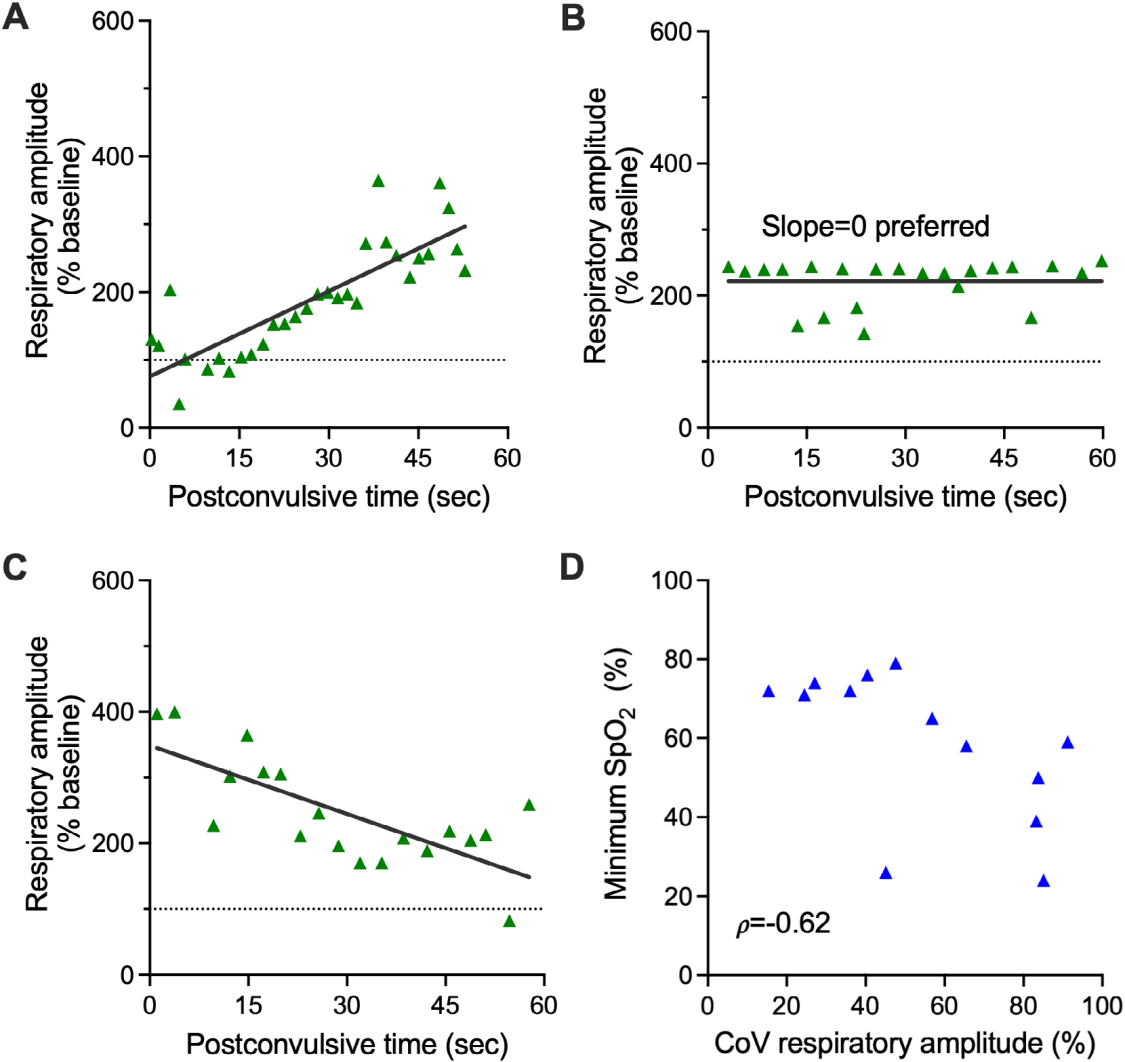
The effects of seizures on respiratory amplitude. A–C show examples of temporal trends in respiratory amplitude from different seizures. Each figure shows the best‐fit line and breath‐by‐breath respiratory inductance plethysmography (RIP) data (green *triangles*) for a single seizure during the first 60 seconds of the postconvulsive period. The dotted line on each figure represents baseline mean amplitude (100%). (A) Shallow respirations for approximately 15 seconds followed by tidal volume recruitment. (B) Relatively stable respiratory amplitude during the entire 60‐second period. (C) Slowly decreasing respiratory amplitude. (D) Seizures characterized by greater variability in respiratory amplitude exhibited more severe oxygen desaturation (𝜌 = −0.62, *p* = 0.0027). [Color figure can be viewed at www.annalsofneurology.org]

Overall respiratory amplitude increased during the first 60 seconds of the postconvulsive period to 273.2 (196.1, 448.3)% of baseline (n = 30 GCS). Linear regression was used to map temporal trends in respiratory amplitude. Using this approach, the best‐fit model of respiratory amplitude versus time had a positive slope in 12 seizures, a slope of 0 in 12 seizures, and a negative slope in 4 seizures (Fig 2A–C). Therefore most, but not all, seizures were characterized by either an immediate increase in tidal volume that was maintained during the first minute of the postconvulsive period or by continued tidal volume recruitment throughout this period. Still, 12 of 32 (37.5%) seizures were characterized by 2 or more grossly ineffective respiratory efforts (amplitude less than interictal baseline despite high metabolic demand), termed “shallow breaths.” In 2 seizures from 1 patient, shallow breathing occurred between the end of the generalized convulsive period and the end of the electrographic seizure. In 1 of these cases, many of the shallow breaths recorded from RIP belts coincided with focal clonic activity of the supraclavicular muscle (an accessory muscle of respiration) as the EEG transitioned to predominantly involve the right frontal lobe. It is not clear whether this represented a causal relationship. Shallow breathing was also frequently irregular even when not accompanied by PCCA (see Fig 1).

When present, shallow respiration usually occurred in the immediate postconvulsive period, in close temporal proximity to the development of critical oxygen desaturation. We, therefore, analyzed this relationship to assess the appropriateness of respiratory motor output during a vulnerable period. Seizures with at least 2 postconvulsive breaths shallower than baseline (n = 7) were associated with a lower SpO_2_ nadir 58 (26–65)% versus 72 (65.8–76.8)%, *p* = 0.047 than seizures without this finding (n = 6). Although overall respiratory amplitude during the first minute was unrelated to SpO_2_ nadir (ρ = 0.22, *p* = 0.47, n = 13), the CoV of respiratory amplitude was negatively related to SpO_2_ nadir (ρ = −0.62, *p* = 0.027, n = 13) (see Fig 2D).

### Thoracoabdominal Asynchrony

All seizures in patients with data available from both RIP belts (30/30, 100%) demonstrated in‐phase (parallel) movements of the chest and abdomen during baseline interictal breathing. In the postconvulsive period, a phenomenon was observed with 24 seizures (24/30, 80.0%) in which movement of the thorax and abdomen were out of phase with one another. There were 2 variations of this pattern.

#### Paradoxical Breathing Pattern in a 1:1 Chest‐Abdomen Ratio

**FIGURE 3:**
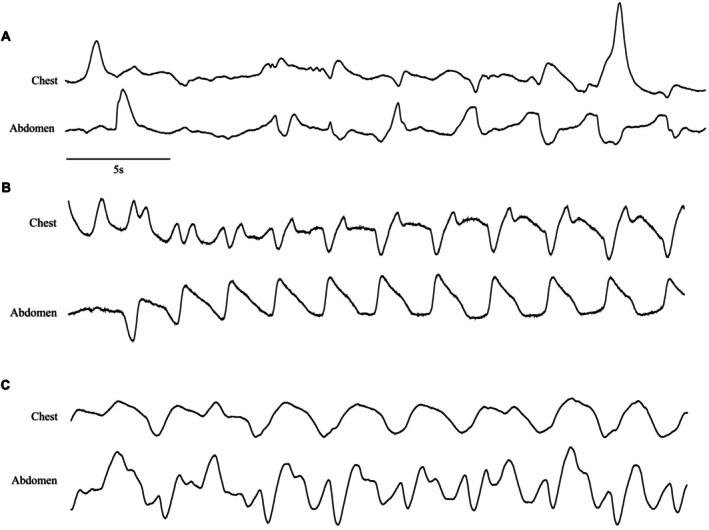
Examples of thoracoabdominal asynchrony. (A) The chest and abdomen are moving in opposing directions while maintaining a 1:1 ratio. Paradoxical movement is a spectrum that can range from complete, in which the chest and abdomen are 100% in opposition, to incomplete in which the chest and abdomen are only partially out of phase. This pattern also demonstrates irregular rhythmicity, which demonstrates the overlap between the different categories of breathing patterns. This patient exhibited a 20mmHg rise in tcCO_2_ postictally. (B) This pattern began with a 2:1 chest:abdomen ratio that gradually evolved into a 1:1 pattern that is directly oppositional. (C) An example of erratic patterning that had a roughly 2:1 ratio of abdomen:chest movement.

In these cases, the chest and abdomen moved in opposite directions, but maintained a 1:1 ratio for the duration of the respiratory cycle. Fifteen seizures (15/30, 50.0%) had evidence of transient or sustained paradoxical breathing during the immediate postconvulsive period (Fig 3A,B). In 3 of these seizures (3/15, 20.0%), paradoxical breathing was noted to substantially improve after re‐positioning, consistent with alleviation of UAO.

Paradoxical breathing tended to persist for longer than shallow breathing, suggesting it may contribute to both the development of hypercapnia and prolonged hypoxemia. However, when compared with in‐phase movement of the chest and abdomen we found no significant relationships between paradoxical breathing and either adequacy of ventilation (tcCO_2_ variables) or severity of oxygen desaturation (SpO_2_ nadir and duration of hypoxemia), although these analyses were limited by small sample sizes (see Data S1).

#### Paradoxical breathing pattern with biphasic or triphasic movement of the chest or abdomen

**FIGURE 4:**
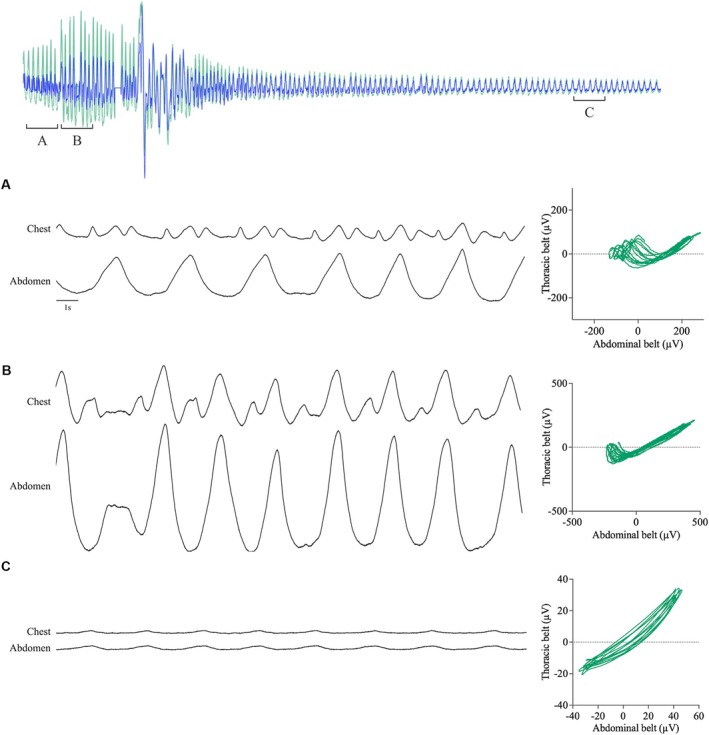
Temporal evolution of an unusual case of thoracoabdominal asynchrony after GCS. The top panel shows changes in chest (blue) and abdominal (green) RIP amplitude over time. The pattern of chest:abdomen movement evolved from (A) triphasic to (B) biphasic to (C) a normal 1:1 pattern. GCS = generalized convulsive seizure. [Color figure can be viewed at www.annalsofneurology.org]

In 16 seizures (16/30, 53.3%), the chest and abdomen moved at different rates for unclear reasons. Of these cases, 13 seizures (13/16, 81.3%) exhibited 2 inspiratory peaks in the chest belts compared to a single inspiratory peak in the abdominal belt (Fig 3B). In 1 of 16 seizures (6.3%) we observed a 3:1 chest to abdomen ratio that decreased to 2:1 and then eventually 1:1, which may also be a manifestation of disordered rhythmicity (Fig 4).

In contrast, 5 of 30 seizures (16.7%) demonstrated biphasic abdominal movement during inspiration compared to a single chest movement (see Fig 3C). One seizure (1/30, 3.3%) first exhibited biphasic inspiratory peaks of the chest belt with a single abdominal excursion, but then transitioned to biphasic inspiratory peaks of the abdominal belt with a single inspiratory peak of the chest belt.

### UAO

UAO was ascertained through a composite analysis of NPT tracings, the identification of 1:1 paradoxical breathing on RIP belts, and noisy upper airway sounds captured on audio‐video recordings, with or without accessory muscle use. Nearly all seizures (30/32, 93.8%) exhibited at least 1 finding consistent with UAO. Even after excluding noisy upper airway sounds as a criterion, evidence of UAO was present in 26 of 32 (81.3%) GCS. As discussed above, 1:1 paradoxical breathing consistent with inspiratory UAO was observed to varying degrees in 15 of 30 seizures (50.0%) (see Fig 3A,B). In 27 of 32 seizures (84.4%), patients exhibited noisy upper airway sounds, both inspiratory (21/32, 65.6%) and expiratory (27/32, 84.4%), the latter associated with grunting or lip fluttering. We also examined the NPT signal for flow limitation compatible with UAO. In 15 of 15 (100%) seizures with interpretable NPT data, waveforms exhibited inspiratory peak flattening consistent with inspiratory flow limitation (Fig S1). In 6 of these cases (6/15, 40.0%), high‐frequency inspiratory (5/15, 33.3%) and/or expiratory (5/15, 33.3%) oscillations were present on NPT tracings, consistent with variable upper airway resistance (see Fig S1). In some cases, UAO improved with repositioning, although the effects of this intervention were difficult to separate from the effects of co‐interventions (ie, stimulation and suctioning).

### Cardiorespiratory Relationships

In 29 GCS, the median instantaneous heart rate during the first minute after the convulsive period ranged from 60.0 to 162.2bpm with a median of 134.8 (123.7–143.8) bpm. Using a 4‐point moving average the median heart rate nadir was 95.6 (81.9–113.4) bpm. Bradycardia occurred in 2 seizures and transient relative bradycardia in 6 (see Data S1).

**FIGURE 5:**
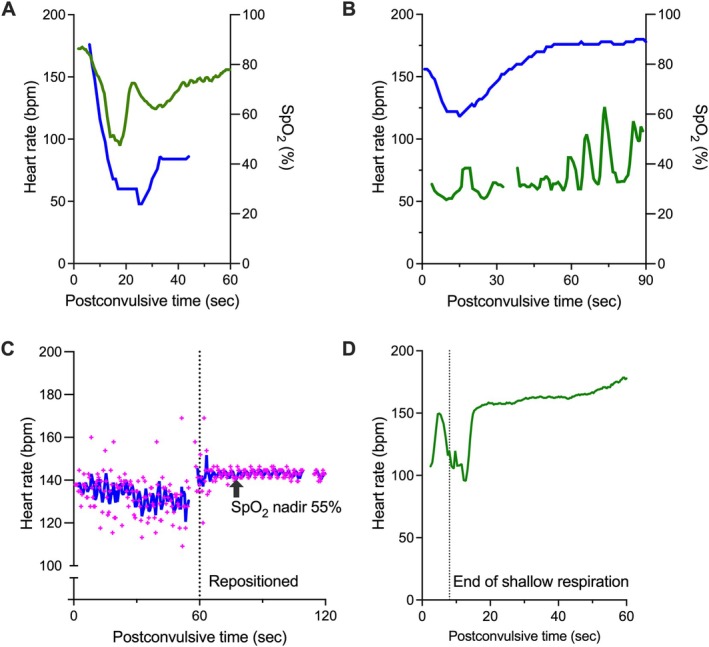
Cardiorespiratory relationships. Each panel shows a different seizure. (A) Transient relative bradycardia (instantaneous heart rate in green) associated with critical oxygen desaturation (SpO_2_ in blue; the signal was lost at 44 seconds). Figure 1 shows respiratory tracings from the same seizure. (B) Bradycardia (4‐point moving average of heart rate in green) associated with shallow and irregular breathing, upper airway obstruction (stertorous breathing, nasal pressure transducer [NPT] flattening), and hypoxemia (SpO_2_ in blue) that gradually improves with time and repositioning. (C) Increased heart rate variability that resolves with repositioning (dotted line), which immediately improved airflow. Magenta stars = instantaneous heart rate, blue line = 4‐point moving average. (D) Relative bradycardia temporally associated with shallow breathing and perioral cyanosis. [Color figure can be viewed at www.annalsofneurology.org]

Median heart rate increased from 72 (60.6–78) bpm interictal to 130.4 (120.1–143.8) bpm postconvulsive period, *p* < 0.0001, n = 21. Patients with absolute (2/21 [9.5%]) or transient relative (6/21 [20.7%]) bradycardia exhibited greater variability in respiratory amplitude than non‐bradycardic patients (CoV respiratory amplitude 74.4 [53.9–89.7]% [n = 8] vs 35.7 [22.9–56.1]% [n = 14], *p* = 0.0197). Bradycardic patients also exhibited longer duration of oxygen desaturation (209 [134.8–233.8] s, n = 4 vs 94.5 [50.8–137.8] s, n = 10, *p* = 0.024) and a non‐significantly lower SpO_2_ nadir (58.0 [31.5–62.0]%, n = 5 vs 72 [55.3–75.5]%, n = 8, *p* = 0.059). There were no significant differences between these 2 groups in median CoV‐IBI or respiratory amplitude (*p* ≥ 0.21) (see Data S1). In some cases, acute changes in heart rate occurred in close temporal proximity to changes in respiratory status (Fig  5).

### Long‐Term Follow‐Up

**FIGURE 6:**
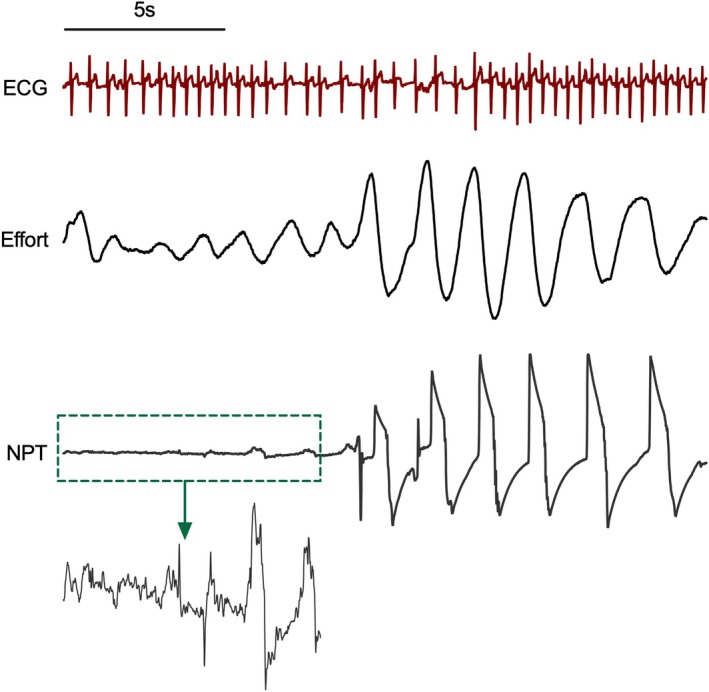
Transient relative bradycardia during the early postictal period. The end of the convulsive period, when the patient is noted to be cyanotic, is marked by the onset of the tracings. Both the chest wall effort sensor and nasal pressure transducer [NPT] tracing exhibit shallow breathing that increases in amplitude over the first 10 seconds. The heart rate slows during the early postconvulsive period, only to increase shortly after respirations increase in amplitude. The same seizure is shown in Figure 5D. [Color figure can be viewed at www.annalsofneurology.org]

Approximately 40 months after EMU admission a male >30 years of age with generalized drug‐resistant epilepsy since childhood and frequent nocturnal GCS died unexpectedly following a witnessed GCS at home. He initially recovered to the point of communicating with emergency medical services. During the subsequent ambulance ride he became anxious, short of breath, and hypoxemic before becoming unresponsive and experiencing a cardiac arrest. An autopsy revealed bilateral proximal pulmonary thromboembolism. The seizure reported in this study was characterized by perioral cyanosis and shallow breathing during the immediate postictal period, as well as by high respiratory amplitude variability, UAO, and relative bradycardia (see Fig 5D and Fig 6 and Supplemental Results for further details). All other patients were living at the time of this report.

## Discussion

This study has several novel findings: (1) GCS frequently induce immediate, severe, non‐apneic respiratory dysfunction and hypoxia. Nearly all GCS were characterized by 1 or more of the following patterns: disordered rhythmicity, shallow breathing, thoracoabdominal asynchrony, or UAO. (2) Postconvulsive breathing that is either shallow or irregular in amplitude is associated with more severe oxygen desaturation. (3) The occurrence of transient or relative bradycardia was associated with greater variability in the amplitude of postictal respiration. Overall, our findings in nonfatal GCS demonstrate that seizures frequently induce respiratory dysfunction severe enough to cause bradyarrhythmias^4^ of the kind witnessed immediately after fatal seizures in MORTEMUS.^2^

Until relatively recently, studies of SUDEP pathogenesis were primarily focused on cardiac etiologies.^27^ There is now accumulating evidence pointing to a primary respiratory defect in many cases of SUDEP.^28^ The current study “connects the dots” between seizure‐induced respiratory and cardiac dysfunction while also showing the former is not limited to apnea. This is important, because in a recent multicenter study showing increased risk of SUDEP in patients with peri‐ictal apnea, ictal and postictal central apnea were only observed in 36% and 43%, respectively, of all SUDEP/near‐SUDEP cases.^29^ This means that the absence of peri‐ictal apnea is not necessarily reassuring, and there is an urgent need to develop more predictive biomarkers of SUDEP risk. We speculate that increased variability in the timing and amplitude of postconvulsive respiration identifies a tendency to seizure‐induced instability of the respiratory network that, in certain contexts, could increase the risk of SUDEP. Of note, prior studies support the potential utility of interictal respiratory variability in predicting severity of postictal oxygen desaturation^11^ and, when measured during non‐rapid eye movement sleep, SUDEP risk.^30^

This study is novel in its comprehensive multimodal assessment of postictal respiration, involving analysis of audiovisual material and tcCO_2_, SpO_2_, airflow, and chest and abdominal RIP signal data by investigators with expertise in respiratory failure. This approach allowed us to characterize respiratory patterns that would have otherwise not been detected by conventional metrics such as respiratory rate. Many of the patterns described above would be classified as having a “normal” respiratory rate, but are markedly abnormal in the context of severe hypoxia and/or hypercapnia. As such, this study demonstrates a variety of respiratory abnormalities in the postictal period, both subtle and overt, that may themselves cause severely decreased ventilation and oxygenation. These findings are, therefore, consistent with those of MORTEMUS,^2^ whose authors described several different patterns of dysfunctional breathing, but because respiration was not directly monitored, were unable to comprehensively characterize them or relate them to oxygenation, ventilation, or the heart rate.

**FIGURE 7:**
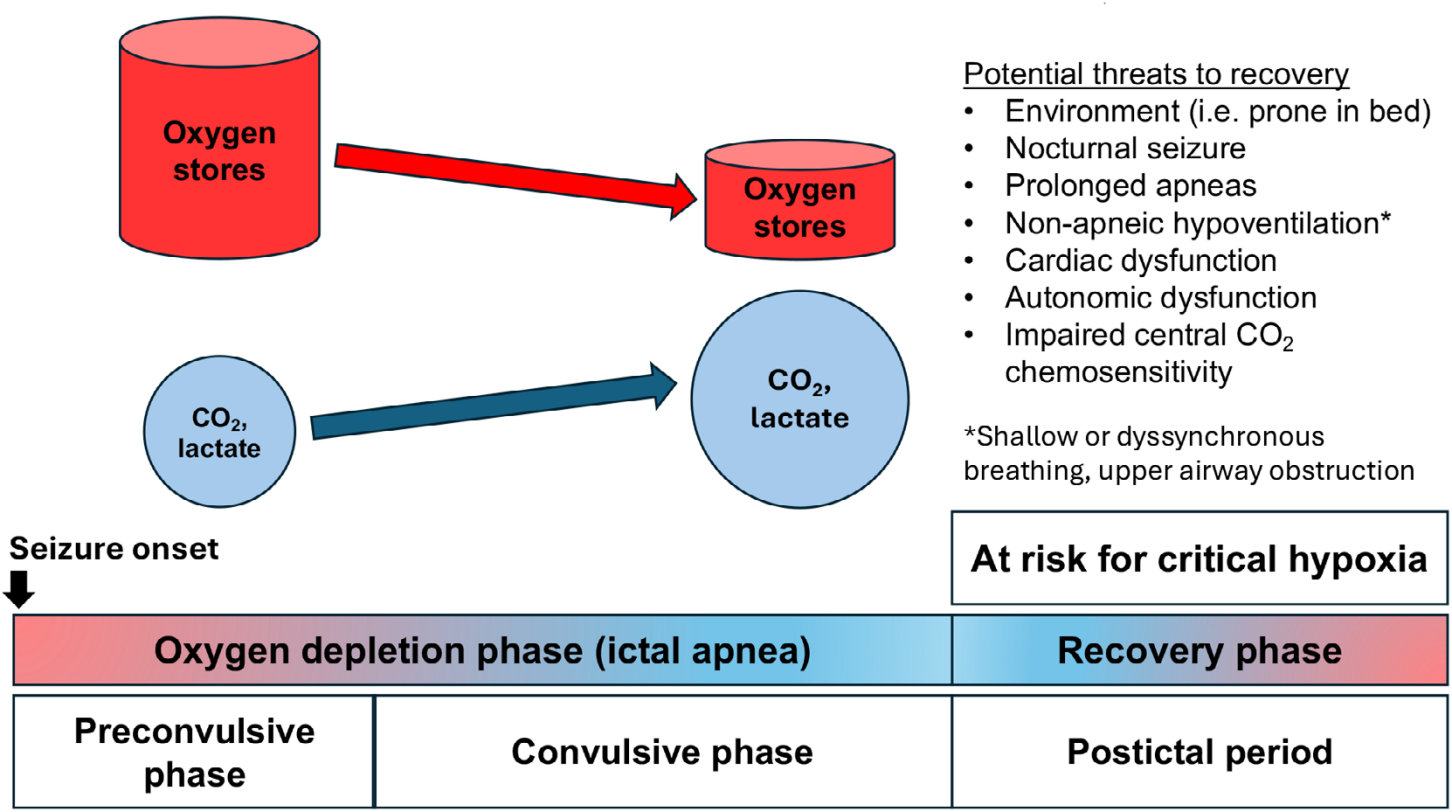
Oxygen depletion and restoration during the peri‐ictal period. The figure illustrates how the combination of ictal apnea and generalized convulsive activity predispose some patients to develop severe hypoxemia during the immediate postictal period. In most cases, postictal ventilation is sufficient to restore oxygenation, but a variety of environmental (ie, prone), contextual (ie, asleep or awake), or physiologic phenomena may interrupt this process. Non‐apneic hypoventilation may result from shallow breathing (typically present during the first 15 seconds of the convulsive phase in nonfatal seizures), dyssynchronous breathing (ie, irregularly timed or patterned), or upper airway obstruction. [Color figure can be viewed at www.annalsofneurology.org]

The metabolic effects of GCS are central to the interpretation of our findings. The forceful muscle contractions of GCS result in an acute CO_2_ load and lactic acidosis.^31^ In a different context, these stimuli would prompt central CO_2_ chemoreceptors to vigorously stimulate ventilation^32^ to restore homeostasis. Adequate ventilation is particularly important in the immediate postictal period because respiratory efforts during the tonic and clonic phases are frequently absent or ineffective, which together with the high metabolic demand from GCS causes oxygen stores to be depleted and predisposes the patient to critical hypoxia. The SpO_2_ nadir in our study occurred a median of only 8 seconds after the convulsive period ended, consistent with ictal apnea being the primary contributor to hypoxemia during the immediate postictal state (Fig 7) and providing an additional stimulus to increase ventilation. In our study, however, breathing was frequently shallow, erratic, and less effective during the first minute postictally despite severe hypercapnia, which is consistent with the occurrence of seizure‐induced depression of central CO_2_ chemoreceptors in some individuals.^13^, ^33^ A failure to adequately respond to hypoxemia and hypercapnia may make patients vulnerable to harsh downstream effects: a cascade of failures that may present simply as bradycardia,^4^ but may also cause a critical reduction in cardiac contractility^34^, ^35^ and hypoperfusion of the brainstem and respiratory muscles. In this sequence, what began primarily as respiratory dysfunction and critical hypoxia leads ultimately to secondary cardiac dysfunction, terminal apnea, and cardiac arrest.

One patient in our study later died after a GCS, with the autopsy revealing bilateral proximal pulmonary thromboembolism. This death could be classified as SUDEP Plus, given that death “may have been due to the combined effect of both conditions.”^36^ Because the chain of events leading to death is not known, it is unclear whether the features of this patient's peri‐ictal physiology described in this report (see Fig 6) are relevant to his death.

UAO was a common finding in our cohort, affecting approximately 90% of all seizures. Postictal UAO is a well‐known complication of GCS and has been highlighted by other investigators.^4^, ^16^ Our definition of UAO was composite and included examination of the NPT and RIP belt signal data and audiovisual review. Expiratory UAO in the form of grunting or lip fluttering was common, and its clinical significance is unclear. Inspiratory UAO is likely of greater importance, given that it causes hypoxemia and sometimes hypercapnia in otherwise normal patients with OSA. Upper airway dilator muscles receive tonic and phasic drive from the respiratory network, and this output is reduced during sleep and when consciousness is diminished, such as during conscious sedation and anesthesia. Audiovisual evidence of UAO is also apparent on our review of a recently published report of a patient who died of SUDEP while undergoing home video‐EEG monitoring.^37^ Upper airway tone is partly mediated by motor output enhanced by serotonergic neurons involved in arousal.^38^, ^39^ Therefore, the co‐occurrence of UAO and hypoventilation are linked mechanistically. It is important to consider UAO clinically as supplemental oxygen may not be sufficient, and the patient may instead require repositioning, insertion of a nasopharyngeal airway, or intubation.

Similar to Vilella et al,^16^ we did not observe laryngospasm in our cohort. Laryngospasm has been reported during a focal seizure^40^ and after GCS,^41^ and animal models suggest it may be a cause of some SUDEP cases.^42^ However, the UAO in this study's nonfatal seizures tended to rapidly improve, including sometimes by stimulation and repositioning, features more commonly associated with reduced upper airway tone than laryngospasm. Furthermore, the upper airway sounds in this study tended to be low and coarse, unlike the higher‐pitch inspiratory stridor more characteristic of laryngospasm.

Nonparallel movement of the chest and abdomen is most commonly observed when the upper airway is obstructed, as in OSA. It is, therefore, likely that the cases of 1:1 paradoxical breathing we observed were because of UAO. However, we also observed other forms of thoracoabdominal asynchrony, including instances in which the abdomen and chest appeared to move at different rates. The etiology of these patterns is not clear. In such cases, the work of breathing frequently appeared elevated, suggesting some of these instances of thoracoabdominal asynchrony represented adaptive responses to loaded breathing^43^, ^44^ from acidosis. Acute hypercapnia also reduces diaphragm contractility and endurance time, and we cannot rule out the possibility of diaphragmatic fatigue if this muscle was tonically contracted during the GCS.^5^, ^45^ Therefore, some instances of thoracoabdominal asynchrony may reflect a strength‐load imbalance between the diaphragm and the metabolic load from the GCS. In other cases, the asynchrony may represent dysfunction of respiratory rhythm generation by the brainstem.

Disordered rhythmicity occurred at some point during most seizures. Two seizures exhibited a pattern of “interrupted expiration” (see Fig 1B) that, to our knowledge, has not been described previously in this population. Similar findings, along with marked variability in breathing, have been reported in the immediate period after birth in humans.^46^, ^47^

The ratio of type 1 to type 3 GCS was lower in our study than has been reported elsewhere,^24^ possibly because of inter‐rater variability in assessment or the relatively small sample size. We also reported a lower rate of PCCA in our study than was reported by Ochoa‐Urrea et al.^15^ This may also reflect sampling differences or inter‐rater variability in the identification of very low amplitude breaths (vs apnea).

### Limitations and Strengths

Our study has some limitations. First, many sensors were removed by patients or became dislodged or malpositioned during GCS. To ensure high‐fidelity measurements, we carefully reviewed the signal data and position of all devices and restricted our analyses to only high‐quality recordings. This decreased the power of our study and limited our ability to conduct some analyses. Second, our analyses of RIP data do not permit us to precisely quantify tidal volume and should be considered semiquantitative. Third, although it is likely that various nursing interventions improved the cardiorespiratory status of some patients (see Fig 6C),^48^ our study was not powered to formally assess this effect. Fourth, because the seizures in this study were nonfatal, we were unable to directly observe cardiorespiratory changes leading to death. Finally, because this was an exploratory study we did not adjust our analyses for multiple comparisons.^49^ This approach increases the risk of a false discovery. Therefore, the inferences made from our statistical tests should be considered hypothesis‐generating, albeit in many cases highly plausible biologically, and should be replicated in a larger cohort.

There are also strengths to this study, including our comprehensive approach to analyzing respiration using metrics not routinely measured in other studies. In every case, video and signal data from the postictal period were reviewed multiple times by the investigators to analyze and integrate multiple sources of complex data. This approach allowed us to characterize the adequacy of ventilation in our patients more comprehensively than prior studies that relied solely on measurements of respiratory rate and oxygen saturation and the visual identification of central apneas. It also permitted the association between dysfunctional breathing and heart rate to be analyzed, highlighting the interdependence of cardiac and respiratory function in the postictal state.^22^

## Conclusions

The results of this study show that GCS frequently cause immediate and severe respiratory dysfunction and hypoxia in the absence of frank apnea, and this respiratory dysfunction is closely associated with changes in heart rate. This suggests some of the immediately postictal bradyarrhythmias observed in MORTEMUS may have been caused by critical hypoxia that occurred before any significant respiratory dysfunction was detected using visual observation and EEG analysis. These data will also guide the identification of high‐risk patients who might require different approaches to the prevention of SUDEP. Defining the sequence of events leading from seizures to death will require direct measurements of all relevant pathological variables, including CO_2_, O_2_, efficacy of alveolar ventilation, and ECG.

## Author Contributions

H.E.P., R.K.S., G.B.R., and B.K.G. contributed to the conception and design of the study; H.E.P., R.K.S., R.N., D.N.D., E.B., L.V., X.L., S.L., G.B.R., and B.K.G. contributed to the acquisition and analysis of data; H.E.P., R.K.S., R.N., E.B., G.B.R., and B.K.G. contributed to drafting the text or preparing the figures.

## Potential Conflicts of Interest

H.E.P., R.K.S., R.N., D.N.D., E.B., G.B.R., and B.K.G. report the SenTec Transcutaneous Monitoring System was provided by the company. Detailed declarations are provided.

## Supporting information


**Data S1.** Supporting Information.

**Figure S1.** Upper airway obstruction evident on NPT tracing. Flattening of the inspiratory peak and high‐frequency oscillations in both inspiratory and expiratory phases is demonstrated. NPT = nasal pressure transducer.

## Data Availability

Some of these data are archived in the Center for SUDEP Research database https://sudepresearch.org. Other de‐identified data will be made available via Epilepsy. Science at https://docs.pennsieve.io/docs/epilepsyscience-1 or by the corresponding author, on reasonable request.
