# Supplementary material for: Severe, Non‐apneic Respiratory Dysfunction and Hypoxia following Generalized Convulsive Seizures
Source: Ann Neurol. 2026 Jan 27;99(5):1263–76. doi: 10.1002/ana.78164 (PMC12885573; doi:10.1002/ana.78164)
Supplement: Supplementary file 1 — Data S1. Supporting Information. [file ANA-99-1263-s002.docx]

Supplementary methods

Study design and participants

Demographic and clinical variables included age, sex, seizure type(s), duration of epilepsy, body mass index (BMI), history of obstructive sleep apnea (OSA), and use of selective serotonin reuptake inhibitors or serotonin-norepinephrine reuptake inhibitors.

Video-electroencephalographic and cardiorespiratory monitoring

EEG data acquisition was performed using a Nihon Kohden system with a sampling rate of 1000 Hz. Chest and abdominal respiratory inductance plethysmography (RIP) belts (Pro-tec zRIP DuraBelt, Philips Respironics) were used to measure effort and assess synchrony between the thoracic and abdominal compartments. Airflow was measured with a nasal pressure transducer (NPT; BiNaps, Salter Labs) and oronasal thermistor (Thermisense, Salter labs). Capillary oxygen saturation (SpO2) and transcutaneous CO2 (tcCO2) were measured on the forehead or cheek (SenTec Transcutaneous Monitoring System) or the fingertip (SpO2 only, Nihon Kohden). The belts’ tendency to slip in some patients, the unpredictable timing of GCS, and the effect of the convulsive activity itself on belt position can result in some recordings being uninterpretable. To minimize this occurrence, all patients were instructed in the use and appropriate positioning of the monitoring devices by trained EEG technicians, who worked with EMU nurses to ensure high-quality recordings and to troubleshoot problems as they arose.

All EEG and cardiorespiratory data were time-synchronized with the video recording. Visual analysis of baseline (interictal) breathing was first performed to confirm regularity of breathing on NPT and RIP belts. Postictal respiratory data were analyzed from the end of the convulsive phase to clinical arousal and/or on return to baseline breathing pattern. To minimize the potential for bias, breathing patterns were characterized before analyzing their relationships to tcCO2 and SpO2.

Onset of oxygen desaturation was defined as the time when SpO2 fell below 90%. Duration of oxygen desaturation was defined as the time from onset of oxygen desaturation to the point of returning to ≥90%. Duration of postictal tcCO2 rise was defined as the time interval between the point at which the tcCO2 increased by ≥10% over the preictal baseline value and the point at which the tcCO2 fell below this value. Magnitude of tcCO2 rise was calculated as the difference between the peak postictal tcCO2 and the preictal baseline tcCO2.

Quantitative analyses of respiratory variability and amplitude were performed on NPT and/or thoracoabdominal RIP signal data using LabChart Pro (ADInstruments, Australia). For these analyses, belts were preferred over NPT, and the belt with the most normal movement was selected. The peak detection tool was used to detect inspiratory peaks. The sensitivity of the tool was manually adjusted to capture all breaths, which were all verified by visual inspection.

For the purposes of this study, the first interbreath interval (IBI) during the postconvulsive period was calculated as follows:

We reported the IBI between the first postconvulsive breath and the last breath from the convulsive period if such a breath was readily identifiable.

If no such breath could be identified, the IBI was calculated by subtracting the time of the first postconvulsive breath from the time convulsions ended (i.e. t=0).

The amplitude of each breath was expressed as a percentage of baseline amplitude. This baseline was calculated as the average amplitude of four consecutive artifact-free breaths from the first period of quiet wakefulness immediately preceding the GCS. Changes in respiratory amplitude over time were modeled using linear regression and the least squares method (x=time, y=amplitude). Akaike’s Information Criteria (AICc) was used to determine whether the slope of the resulting model or a model with a slope of zero best fit the data.

The baseline interictal heart rate was calculated from the first artifact-free 10-second segment beginning one minute prior to seizure onset. R-R intervals (RRI) were calculated using the LabChart Pro Heart Rate Variability module (ADInstruments, Australia), with every heartbeat verified manually. If a sample included more than 8 continuous seconds of missing data, the entire sample was discarded.

Supplementary results

Seizure characteristics

In six seizures, belt malposition made interpretation of the RIP signal impossible. Four seizures were excluded for poor quality RIP signal from a combination of belt malposition and movement artifact from nursing care activities.

In analyses restricted to the first evaluable seizure, SpO2 nadir was not associated with vigilance state at seizure onset (59 [39-72] % awake [n=7] vs 71.5 [43.5-74.5] % asleep [n=6], p=0.66) or duration of PGES (𝜌=0.078, p=0.80, n=13). There were 3 seizures with postictal apnea, 2 of whom had evaluable SpO2 nadirs. These nadirs occurred 25 and 6 seconds after the end of the convulsive period.

Thoracoabdominal asynchrony

Paradoxical breathing tended to persist for longer than shallow breathing, suggesting it may contribute to both the development of hypercapnia and prolonged hypoxemia. However, when compared with in-phase movement of the chest and abdomen we found no significant relationships between paradoxical breathing and either peak tcCO2 (56 [54.3-60.3] mm Hg paradoxical [n=8] vs 64 [45-69] mm Hg in-phase [n=7], p=0.41 or duration of tcCO2 elevation (538.5 [281.3-698.3] s paradoxical [n=8] vs 328.5 [200-577.8] s in-phase [n=8], p=0.36. Paradoxical breathing was also not associated with SpO2 nadir (58 [32.5-75] % paradoxical [n=5] vs 71 [59-72] % in-phase [n=7], p=0.72, or duration of oxygen desaturation (170 [108-197] s paradoxical [n=7] vs 97.5 [49.8-142.3] s in-phase [n=6], p=0.23.

Cardiorespiratory relationships

Interictal baseline heart rates for the two patients with postictal bradycardia were 72 and 73, and for the six subjects with transient relative bradycardia were 102, 72, 60, 63, 78, and 63.

Median respiratory amplitude was not different between patients with absolute or transient relative bradycardia versus those without bradycardia, 211.4 (186.6-294.7), n=8 vs 288.3 (211.3-448.3), n=14, p=0.21. CoV-IBI was also not different between these two groups, 30.5 (21.0-50.6) %, n=8 bradycardia vs 23.4 (18.1-38.1) %, n=14 without bradycardia, p=0.30. Heart rate nadir was not associated with CoV-IBI (𝜌=-0.24, p=0.29, n=21) or CoV respiratory amplitude (𝜌=-0.31, p=0.17).

Long-term follow-up

The nonfatal seizure experienced by the decedent that is described in this study was a type 1 GCS with generalized onset during wakefulness, lasting 79 seconds and with a convulsive period lasting 51 seconds. PGES lasted 6 seconds. At the time of the seizure the patient was being monitored with 3-lead ECG, NPT and oronasal thermistor, SpO2 and transcutaneous CO2, and a single piezoelectric effort monitor on the upper chest (no RIP belts). Lapses in the SpO2 signal precluded SpO2 nadir determination but perioral cyanosis was verbally reported by nursing two seconds prior to the end of the convulsive period and noted to be improving 41 seconds later. Postictal breathing was characterized by initially shallow respirations, as well as by high respiratory amplitude variability, noisy inspiratory and expiratory upper airway sounds, NPT flattening, and relative bradycardia (Fig. 6D). All nursing during the first postictal minute was administered with the patient in the supine position.

Postconvulsive time (sec) Event

-4 Suctioning

-2 Lips noted by nursing to be light blue

0 End of convulsive period

10 Suctioning

35 Oxygen by nasal cannula applied

39 Noted he was starting to “pink up”

49 Withdraws from suctioning, tries to sit up with eyes open

The tcCO2 rose from 36 to 47 mm Hg and was elevated over baseline for 268 seconds.
