# Supplementary figures and images for: Severe, Non‐apneic Respiratory Dysfunction and Hypoxia following Generalized Convulsive Seizures
Source: Ann Neurol. 2026 Jan 27;99(5):1263–76. doi: 10.1002/ana.78164 (PMC12885573; doi:10.1002/ana.78164)

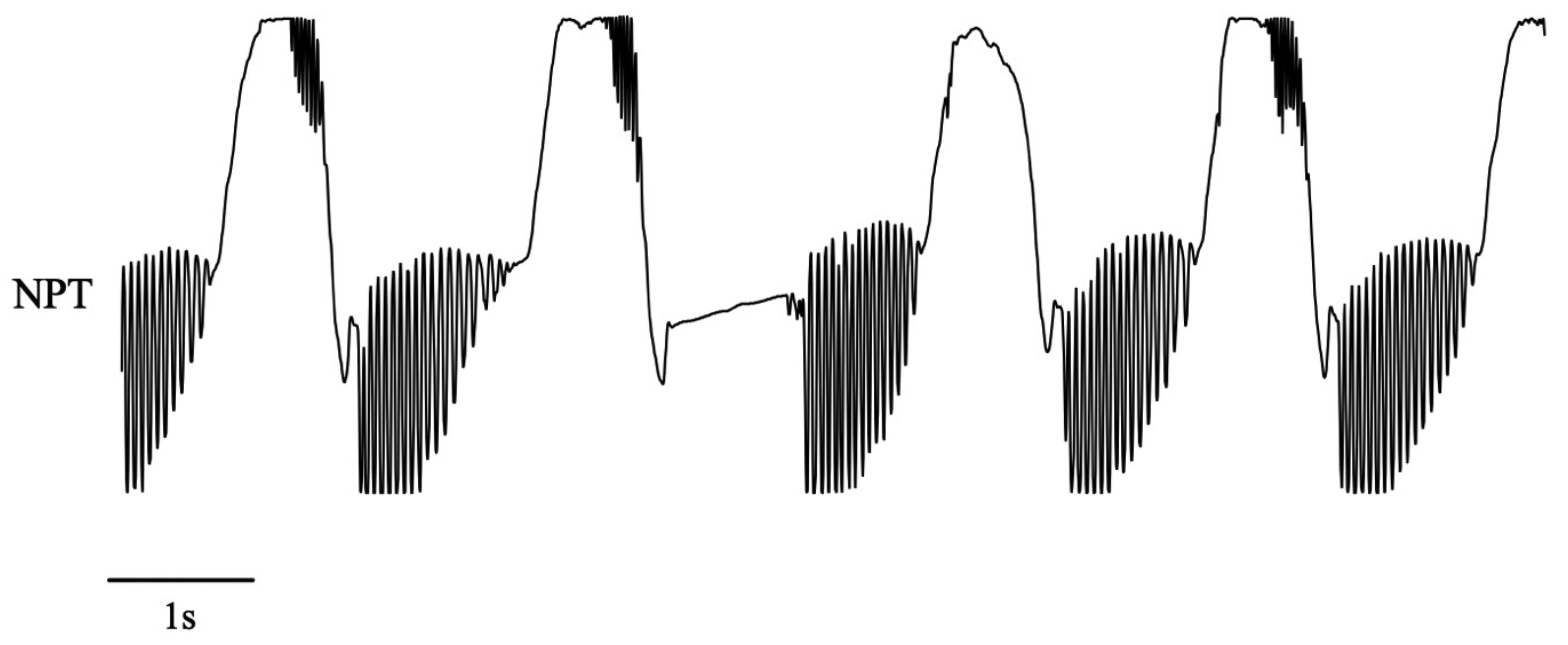

Supplement: Supplementary file 2 — Figure S1. Upper airway obstruction evident on NPT tracing. Flattening of the inspiratory peak and high‐frequency oscillations in both inspiratory and expiratory phases is demonstrated. NPT = nasal pressure transducer. [file ANA-99-1263-s001.tif]
